# Attention-Induced Variance and Noise Correlation Reduction in Macaque V1 Is Mediated by NMDA Receptors

**DOI:** 10.1016/j.neuron.2013.03.029

**Published:** 2013-05-22

**Authors:** Jose L. Herrero, Marc A. Gieselmann, Mehdi Sanayei, Alexander Thiele

**Affiliations:** 1 Institute of Neuroscience, Newcastle University, Newcastle upon Tyne, NE2 4HH, UK

## Abstract

Attention improves perception by affecting different aspects of the neuronal code. It enhances firing rates, it reduces firing rate variability and noise correlations of neurons, and it alters the strength of oscillatory activity. Attention-induced rate enhancement in striate cortex requires cholinergic mechanisms. The neuropharmacological mechanisms responsible for attention-induced variance and noise correlation reduction or those supporting changes in oscillatory activity are unknown. We show that ionotropic glutamatergic receptor activation is required for attention-induced rate variance, noise correlation, and LFP gamma power reduction in macaque V1, but not for attention-induced rate modulations. NMDA receptors mediate attention-induced variance reduction and attention-induced noise correlation reduction. Our results demonstrate that attention improves sensory processing by a variety of mechanisms that are dissociable at the receptor level.

## Highlights

• NMDA receptors aid variance and covariance reduction
• Cholinergic and NMDA receptors serve different functions in attentional modulation
• Attention affects second-order statistics of neuronal activity in V1
• NMDA and AMPA receptor blockade oppositely affect V1 gamma frequency oscillations

## Teaser

Attention improves neuronal processing by increasing response strength and fidelity. Herrero et al. show that NMDA receptor availability is selectively required for increased response fidelity. The study isolates attentional mechanisms at the receptor level.

## Introduction

**Figure 1:**
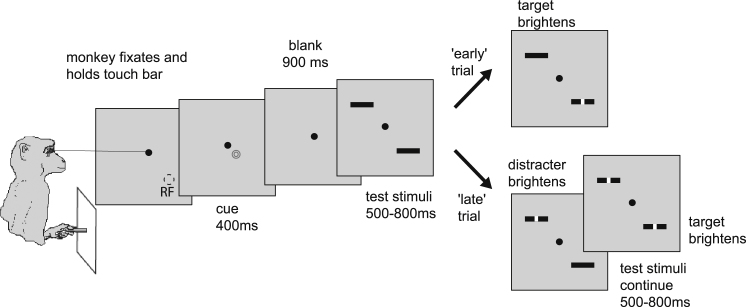
Behavioral Paradigm Monkeys had to fixate and hold a touch bar. Thereupon, a cue appeared that indicated where to attend to (in the example, the animal would have to attend to the stimulus within the receptive field). Following a gap period of 900 ms, two stimuli were presented, one in the receptive field of the neuron under study and another in the opposite hemifield. The animal had to detect a luminance change in the cued location and ignore luminance changes in the uncued location. Task timing is indicated below and above the panels. The monkey had to fixate the fixation point throughout the entire trial.

Selective attention improves sensory processing by altering firing rates, rate variance, and rate covariance in visual cortex (Cohen and Maunsell, 2009; Mitchell et al., 2007, 2009; Moran and Desimone, 1985; Roberts et al., 2007; Roelfsema et al., 1998; Spitzer et al., 1988; Treue and Maunsell, 1996). All of these alterations can improve the signal-to-noise ratio when decoding the activity from single neurons or from pools of neurons (Cohen and Kohn, 2011; Mitchell et al., 2009; Shadlen and Newsome, 1998). Attention equally alters neuronal oscillations in different frequency bands, but the sign of these effects can differ between tasks (Lakatos et al., 2008; Schroeder and Lakatos, 2009), cortical areas (Chalk et al., 2010; Fries et al., 2001), and between cortical layers (Buffalo et al., 2011). Attention-induced firing rate modulations of V1 neurons depend on cholinergic mechanisms (Herrero et al., 2008), which may enable feedback from higher areas to exert its influence (Deco and Thiele, 2011). The mechanisms underpinning rate variance alterations, covariance alterations, or changes in oscillatory activity are unknown. Feedback from higher areas is key for attentional signals to affect sensory processing (Buschman and Miller, 2007; Gregoriou et al., 2009; Moore and Armstrong, 2003; Noudoost and Moore, 2011; Roelfsema et al., 1998; Ruff et al., 2006) and has been proposed to terminate preferentially on N-methyl-D-aspartic acid receptor (NMDA)-rich synapses (Self et al., 2012; Shima and Tanji, 1993, 1998). The precise contribution of NMDA receptors to attentional control is currently unclear, but its involvement in cognitive function has often been emphasized (Brunel and Wang, 2001; Compte et al., 2000; Rowland et al., 2005; Self et al., 2012; Soltani and Koch, 2010; Turchi and Sarter, 2001). NMDA receptors aid coincidence detection. They require postsynaptic depolarization to ensure relief from Mg^2+^ blockade and simultaneous presynaptic activity for glutamate-induced activation. Thus, if feedback from higher areas targeted NMDA receptor-rich synapses, the effect of feedback would be mostly visible if neurons were also driven by sensory input, as has been reported regularly (Luck et al., 1997; Roberts et al., 2007; Treue and Maunsell, 1996). To shed light on this contribution, we investigated how NMDA receptors affect different coding schemes involved in attentional control and contrast this with the contribution of AMPA/kainate receptors. We combined pharmacological analysis of ionotropic glutamatergic receptors (IGluR: NMDA or AMPA/kainate receptors) with single-cell recordings in V1 of behaving macaque monkeys, using task (Figure 1), neurophysiological, and surgical procedures as previously described (Herrero et al., 2008; Thiele et al., 2006). Subjects performed a task demanding allocation of top-down spatial attention toward the receptive field (attend RF) and away from it (attend away) under control conditions and when NMDA (NMDA receptor agonist), DL-2-amino-5-phosphonopentanoic acid (APV: NMDA receptor antagonist), or 6-cyano-7-nitroquinoxaline-2,3-dione (CNQX: AMPA-/kainate receptor antagonist) were iontophoretically applied in the immediate vicinity of the recorded neurons (Experimental Procedures). Neurons were activated by a bar stimulus of optimal orientation centered on their receptive fields (RF), and the locus of attention was manipulated by briefly presenting a visual cue toward or away from the neurons’ RFs before stimulus onset. Monkeys were required to detect and report a subtle change of the stimulus contrast at the attended location and ignore changes at the unattended location, while fixating a central fixation spot (see the Experimental Procedures for additional information).

## Results

We recorded 451 neurons in two monkeys (203 in monkey 1 and 248 in monkey 2) in the presence and absence of different glutamatergic antagonist/agonist. Neurons were included in the current paper if the activity was stable during the different no-drug/drug conditions with good recovery from drug-applied conditions (see the Experimental Procedures for details). APV was tested in 207 neurons, NMDA was tested in 87 neurons, and CNQX was tested in 157 neurons. Of the 451 cells recorded, 221 passed the neuronal exclusion criteria (outlined in detail in the Experimental Procedures). We included 82/207 neurons that were tested with and without APV applied. Of these, 75 were significantly affected by the drug (p < 0.05, two-factor ANOVA, see the Experimental Procedures). We included 96/157 neurons that were tested with and without CNQX applied. Of these, 89 were affected by the drug (p < 0.05, two-factor ANOVA, see the Experimental Procedures). We included 43/87 neurons that were tested with and without NMDA applied. Of these, 40 neurons were affected by application of NMDA (p < 0.05, two-factor ANOVA, see the Experimental Procedures).

Because differential eye movements in attention or drug studies are often a concern, we analyzed eye position for the different attention and drug conditions for the sample of neurons reported above. We have previously demonstrated that tiny residual eye movements were not responsible for the attentional modulation seen in area V1 (Herrero et al., 2008; Roberts et al., 2007) or for the cholinergic drug effects (Herrero et al., 2008). However, to determine whether this also applies to our current data set, we calculated the mean eye-position (x- and y-position) from 200 ms after stimulus onset until 500 ms after stimulus onset in each trial for each condition (attend RF versus attend away; no-drug versus drug applied). This time period was chosen as it was also the time period used to determine effects of attention and of drug application on neuronal activity. We neither found a significant main effect of attention (p > 0.15, two-factor ANOVA), nor a significant main effect of drug (applied versus not applied, p > 0.3, two-factor ANOVA), nor did we find a significant interaction between attention and drug (p > 0.2, 2 factor ANOVA) in any of our data sets (NMDA, APV, or CNQX; eye position for the different data sets were analyzed separately), i.e., neither the locus of attention nor drug application significantly affected the x- or y-eye position.

### Basic Effects of Attention and APV, CNQX, and NMDA on Firing Rates

**Figure 2:**
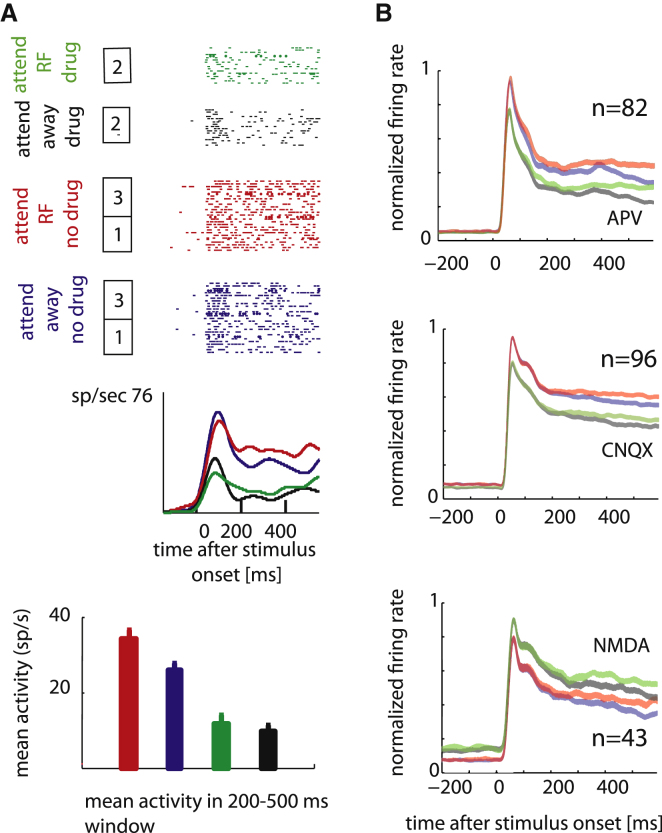
Effect of Drug Application on Firing Rates (A) Effect of attention and APV application on example single cell responses. Raster plots show trial wise spiking activity for the four different conditions (attend RF no drug, red; attend away no drug, blue; attend RF drug, green; attend away drug, black; color code applies to all subplots). The mean activity (+SEM) for the time period of 200–500 ms after stimulus onset is shown below the raster plots and peristimulus time histograms. The numbers in the boxes next to the raster plots indicate trial order, i.e., 1 = initial recording, 2 = block of trials when APV was applied, and 3 = recovery period. (B) Normalized population activity for the four different conditions when APV was not applied and applied (top row), when CNQX was not applied and applied (middle row), and when NMDA was not applied and applied (bottom row). The width of the color-coded histograms indicates mean ± SEM. n, number of neurons.

Figure 2A shows an example of a cell tested with and without APV applied. The cell showed significant attentional modulation during the response period from 200–500 ms after stimulus onset (p < 0.001, two-factor ANOVA), and the application of APV significantly reduced firing rates (p < 0.001, two-factor ANOVA). There was no interaction between attention and drug applied for the cell shown (p > 0.05, two-factor ANOVA). The effects of the different drugs on population firing rates are shown in Figure 2B. The figure shows normalized population histograms for the two attention and the drug conditions for our APV, CNQX, and NMDA samples, respectively.

Attention to the receptive fields of the neurons significantly increased the neuronal activity for our population of cells (p < 0.001, signed-rank test; the nonnormalized population activity is listed in Table 1). APV and CNQX application significantly decreased the population activity (p < 0.001, signed-rank test; Table 1), whereas NMDA application significantly increased the population activity (p < 0.001, signed-rank test; Table 1). Overall the rate reduction induced by CNQX was slightly larger than that induced by APV (Table 1). To determine whether the effect of CNQX was significantly different from the effect induced by APV, we calculated a drug modulation index (MI_drug_), which quantified the effect of the drug in the attend away condition (MI_drug_ = [attend _away no drug_ − attend _away drug_)/ [attend _away no drug_ + attend _away drug_]. The MI_drug_ distribution in the APV condition had a lower median than the distribution in the CNQX condition, but the effect did not quite reach significance (p = 0.07, rank sum test), demonstrating that differences were overall moderate.

### Interaction of Attention and Drug Application on Firing Rates

**Figure 3:**
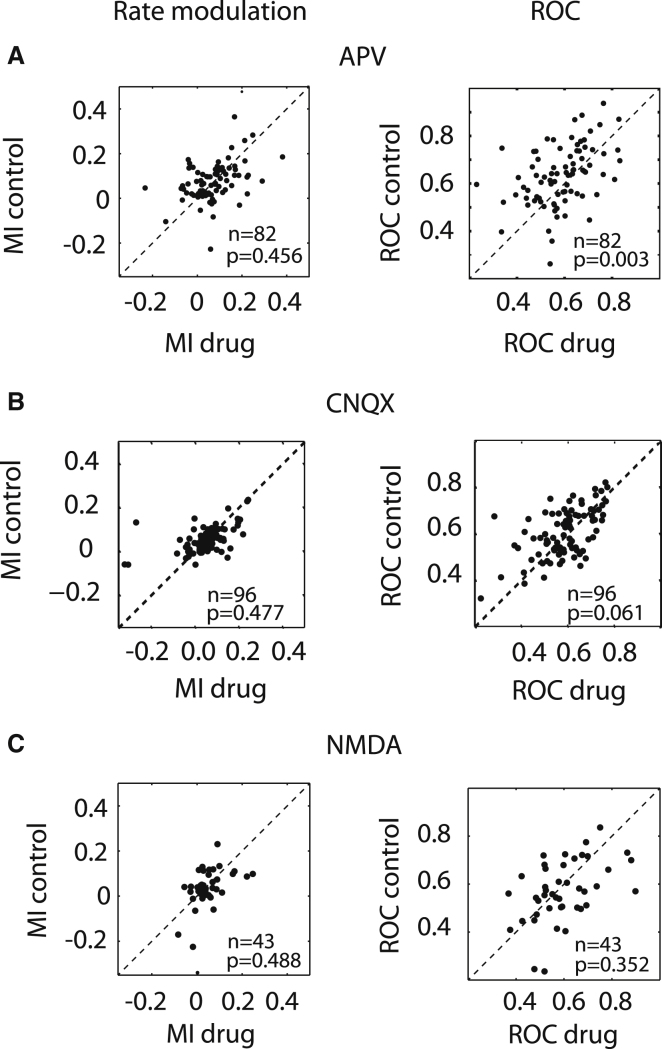
Distribution of Attentional Rate Modulation Indices and Receiver Operating Characteristics during Control and Drug-Applied Conditions (A) Effect of NMDA receptor blockade on attentional rate modulation indices (MI, left column) and on receiver operating characteristics values (ROC, right column). (B) Effect of AMPA/kainate receptor blockade on attentional rate MI (left column) and on ROC values (right column). (C) Effect of NMDA receptor activation on attentional rate MI (left column) and on ROC values (right column). p values indicate whether drug application significantly affected MIs or ROCs (signed rank test). n = sample sizes.

The effects of drug application on the attentional rate modulation for the population of neurons were quantified by calculating an attentional rate modulation index (MI = [attend RF_rate_ − attend away_rate_]/[attend RF_rate_ + attend away_rate_]) and by calculating a receiver operating characteristic (ROC), a nonparametric measure of how well an ideal observer can detect where an animal attends to, based on single trial firing rates. Attentional rate modulation indices were not significantly affected by blockade of NMDA receptors (Figure 3A, p = 0.456, Wilcoxon signed-rank test), whereas ROCs were affected by APV application (p = 0.003, Wilcoxon signed-rank test). Blocking AMPA/kainate receptors (Figure 3B) did not affect attentional rate modulation indices (p = 0.477, Wilcoxon signed-rank test). There was a trend toward reducing ROC values (p = 0.061, Wilcoxon signed-rank test) when CNQX was applied. Application of NMDA itself did not alter either measure (Figure 3C), even though NMDA increased firing rates significantly overall (p < 0.001, signed-rank test; Table 1).

### Interaction of Attention and Drug Application on Rate Variance

**Figure 4:**
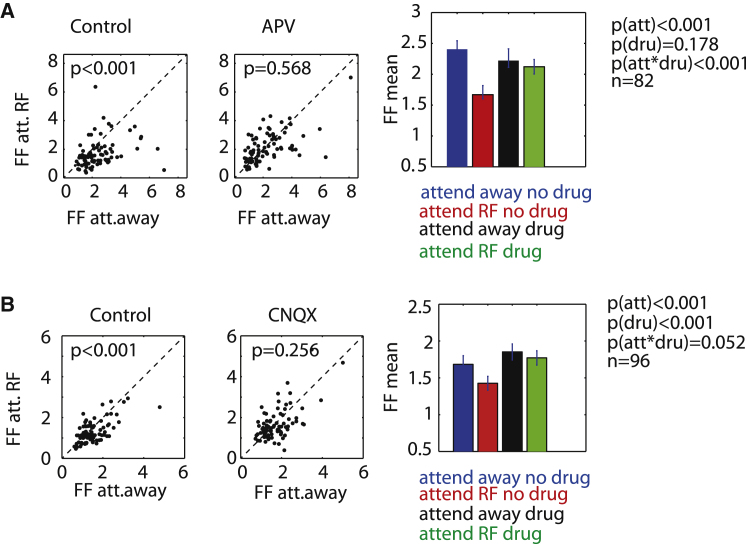
Distributions, Means, and SEM for Effects of Attention and Drug Application on Fano Factors (A) Fano factor (FF) distribution for the attend away and attend RF condition in the absence (left) and presence (middle) of APV. Right bar graphs show mean of the respective distributions and SEM. (B) As in (A) but when CNQX was applied. p values indicate whether drug application significantly affected FFs (ANOVA and signed-rank test [for post hoc testing], respectively). n = sample sizes. See also Figure S2.

How can the discrepancy between drug effects on MI and ROCs arise? ROCs take the mean and the variance of two response distributions into account, whereas MI only takes differences in mean firing rates into account. Given that MI was not affected by drug manipulations, whereas ROCs were, attention and ionotropic glutamate receptor (IGluR) blockade appeared to differentially affect firing rate variance. We tested this by calculating the Fano factor (FF = variance/mean) for the attend RF and attend away condition during control and drug-applied trials. Attention significantly reduced the FF in the absence of drug application (Figures 4A and 4B, p < 0.001 Wilcoxon signed-rank test), an effect previously reported for neurons in area V4 (Mitchell et al., 2007) and MT (Niebergall et al., 2011), but not yet for neurons in V1. However, when NMDA receptors were blocked, attention no longer reduced the FF (p = 0.568, Wilcoxon signed-rank test). A two-factor RM-ANOVA supports this conclusion. We found a significant main effect of attention on FF (p < 0.001) and no significant main effect of NMDA receptor blockade on FF (p = 0.178) but a significant interaction between attention and NMDA receptor blockade (p < 0.001). AMPA/kainate receptor blockade had a somewhat similar effect on attention-induced reduction of FF. FFs were significantly reduced by attention in the absence of CNQX, but not in the presence of CNQX (p < 0.001 and p = 0.256, respectively, Wilcoxon signed-rank test). A two-factor RM-ANOVA revealed that the effects of CNQX on attention-induced FF were less pronounced than those seen with APV, as the attention-drug interaction only showed a trend toward significance (drug^∗^attention interaction: p = 0.052, two-factor RM-ANOVA). Despite the above-mentioned similarity, the two drugs nevertheless affected FFs differently (Figure 4). APV application did not increase FFs above the average no drug condition (Figure 4A, main effect of drug: p = 0.178, two-factor RM-ANOVA), whereas CNQX increased FFs overall (Figure 4B, main effect of drug: p < 0.001, two-factor RM-ANOVA) and beyond the range seen in the attend away no drug condition. Stimulating NMDA receptors by application of NMDA did not affect attention-induced FF reduction, and it had no effect on FFs overall (data not shown).

### Interaction of Attention and Drug Application on Noise Correlations

**Figure 5:**
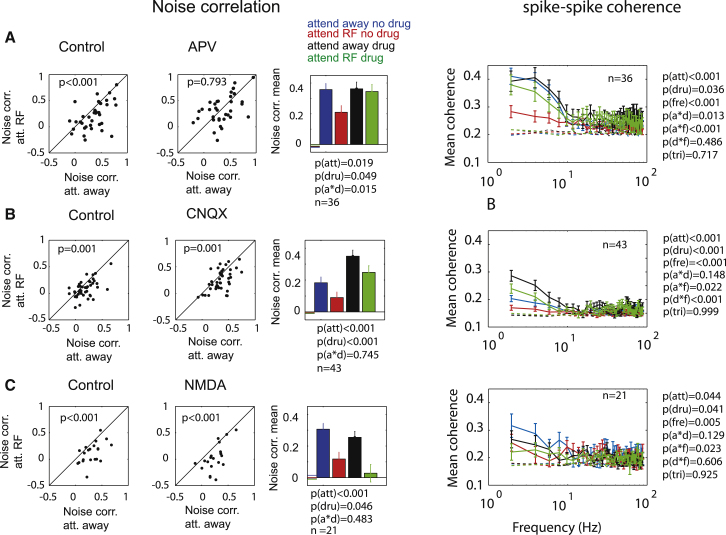
Distributions, Means, and SEM for Effects of Attention and Drug Application on Noise Correlations and Spike-Spike Coherence (A) Noise correlation distribution and spike-spike coherence for the attend away and attend RF condition in the absence (left) and presence (middle) of APV. Right bar graphs show mean of the respective distributions and SEM. (B) As in (A) but when CNQX was applied. (C) As in (A) but when NMDA was applied. p values indicate whether drug application significantly affected noise correlations (ANOVAs and signed-rank tests). n, sample sizes. The small horizontal lines in the right column bar graph (next to the blue bar) show the size of shuffle predictor noise correlations. The right column shows the effects of attention and drug application on spike-spike coherence in different frequency bands. p values indicate whether attention (a), drug application (d), or frequency band analyses (f) significantly affected spike-spike coherence (three-factor ANOVA) or whether a significant interaction existed between these factors (indicated by the respective letters and ^∗^). n = sample sizes. The dashed lines show the size of spike-spike coherence based on shuffled data (shuffle predictor). See also Figures S1 and S3.

Attention reduces noise correlations of simultaneously recorded neurons in macaque V4 (Cohen and Maunsell, 2009; Mitchell et al., 2009). A reduction in noise correlation potentially increases the signal-to-noise ratio, provided a downstream decoder pools the activity from many neurons (Mitchell et al., 2009). We found that attention-induced noise correlation reduction is not restricted to extrastriate cortex but also occurs in V1 (Figure 5, p < 0.001, Wilcoxon signed-rank test). Importantly, blockade of NMDA receptors by application of APV abolished the influence of attention on noise correlations (Figure 5A), evident by a significant interaction between the effects of attention and drug (p = 0.015, two-factor RM-ANOVA). Inspection of the bar graphs in Figure 5A reveals that NMDA receptor blockade selectively eliminated the influence of attention on noise correlation, as noise correlations in the presence of APV were similar to those recorded in the attend-away/control condition. Conversely, AMPA/kainate receptor blockade resulted in overall increased noise correlations (Figure 5B, main effect of drug: p < 0.001, two-factor RM-ANOVA), but it did not abolish the attention-induced reduction of noise correlations (see scatterplots in Figure 5B and bar graphs). A two-factor RM-ANOVA also failed to reveal a significant interaction between attention and drug on noise correlations (attention ^∗^ drug interaction: p = 0.745, two-factor RM-ANOVA). Artificial activation of NMDA receptors by application of NMDA reduced noise correlations overall (Figure 5C, main effect of drug: 0.046, two-factor RM-ANOVA), but it had no effect on the attention-induced reduction of noise correlations (drug ^∗^ attention interaction: p = 0.483, two-factor RM-ANOVA). This shows that availability of NMDA receptors, but not of AMPA/kainate receptors, is critical for attention-induced noise correlation reduction, whereas AMPA/kainate receptor availability is required for an overall reduction of noise correlations, regardless of whether or not attention is directed to the receptive field of the neurons. These results were unaffected when different shift predictor corrections were performed (see the Experimental Procedures; Figure S1 available online).

### Interaction of Attention and Drug Application on Spike-Spike Coherence

An alternative way of (noise) correlation analysis is to determine spike-spike coherence in different frequency bands. Mitchell et al. (2009) analyzed spike-spike coherence in different frequency bands to determine how attention affects correlated activity between simultaneously recorded neurons in area V4. They reported that attention affects spike-spike coherence mostly in lower frequency bands (<10–15Hz). To assess whether this equally applies to neurons in V1, we calculated spike-spike coherence under control conditions and when APV, CNQX, or NMDA were applied (Figures 5A–5C, respectively, right-most column). As reported for area V4, continuous attention reduced spike-spike coherence mostly in lower frequency bands in V1 (see Figures 5A–5C, right column; p values relating to main effects of attention, drug, frequency, and the respective interactions are shown in the figure). Blockade of NMDA receptors strongly reduced the effect of attention on spike-spike coherence, and there was a significant interaction between the effect of attention and the effect of the drug (attention^∗^drug interaction: p = 0.013, Figure 5A). Blockade of AMPA/kainate receptors had very different effects. Overall coherence was increased by drug application (p < 0.001, main effect of drug, three-factor ANOVA), but the influence of attention in reducing coherence was unaffected (attention^∗^drug interaction: p = 0.148, Figure 5B, right column). This is similar to the outcome from our noise correlation analysis. Thus, NMDA receptors are involved in attention-induced coherence reduction, whereas AMPA/kainate receptors are not. Stimulating NMDA receptors directly by application of NMDA reduced spike-spike coherence across low-frequency bands.

### Interaction of Attention and Drug Application on LFP Oscillatory Activity

**Figure 6:**
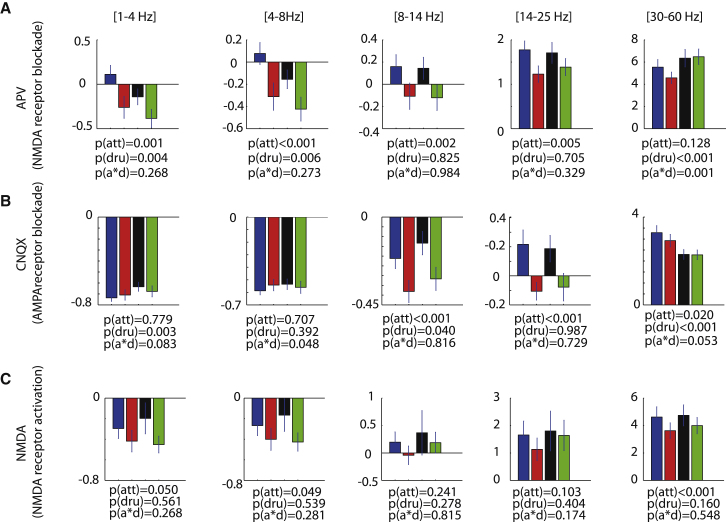
Means and SEM for Effects of Attention and Drug Application on *Z* Score LFP Power in Different Frequency Bands (A) Effect of APV on LFP power (n = 61 experiments). (B) Effect of CNQX on *Z* score LFP power (n = 61 experiments). (C) Effect of NMDA on *Z* score LFP power (n = 28 experiments). p values indicate whether attention or drug application significantly affected any of these measures or whether there was a significant interaction between the two (two-factor ANOVA). See also Figure S4.

In addition to affecting spiking activity within the immediate vicinity of the electrode tip, NMDA receptor blockade affected the overall V1 network as evident from local field potential (LFP) measures. We quantified the LFP delta (1–4 Hz), theta (4–8 Hz), alpha (7–13 Hz), beta (13–25Hz), and gamma (30–60 Hz) frequency power by calculating the *Z* score (see the Experimental Procedures). Attention consistently reduced the LFP power in the gamma frequency range, whereas the effect of attention on LFP power in other frequency bands was more variable (see Figure 6 for details).

NMDA receptor blockade (APV application) significantly reduced the LFP power in the delta and theta frequency range, and it increased the LFP power in the gamma frequency range (main effect of drug: p < 0.05, two-factor ANOVA). AMPA receptor blockade (CNQX application), on the other hand, significantly increased the LFP power in the delta and alpha frequency range, and it decreased the LFP power in the gamma frequency range (main effect of drug: p < 0.05, two-factor ANOVA). Thus, the effect of the two drugs on low and higher frequency ranges was in opposite directions.

Blockade of NMDA receptors by APV abolished the attention-induced reduction of gamma power (attention^∗^drug interaction: p = 0.001, two-factor ANOVA). Blockade of AMPA/kainate receptors by CNQX showed a trend of reducing the effects of attention on LFP gamma power, but the interaction term did not quite reach significance (attention^∗^drug interaction: p = 0.053, two-factor ANOVA).

### Drug Effects on Reaction Times

**Figure 7:**
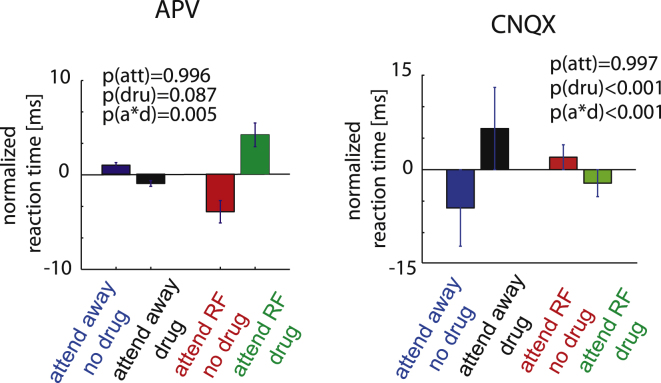
Means and SEM for Effects of Attention and Drug Application on the Animals’ Reaction Times Reaction times are normalized relative to the mean in every session. p values indicate whether attention or drug application significantly affected any of these measures or whether there was a significant interaction between the two (two-factor ANOVA). See also Figure S5.

Given the local, small volume drug application (iontophoretic application), we were surprised to find significant effects of both APV and CNQX on reaction times (RT). Crucially, the effects depended on where the animal was attending to, and they differed between the two drugs. When NMDA receptors were blocked with APV, an animal’s ability to detect the relevant luminance change was only affected during attend RF trials, but not during attend away trials. This was evident by a significant increase in RTs in the attend RF condition, but there was no increase in the attend away condition (drug ^∗^ attention interaction: p = 0.005, two-factor ANOVA, Figure 7). Conversely, when AMPA/kainate receptors were blocked, we found RTs were most strongly affected during attend away trials. AMPA/kainate receptor blockade increased RTs on attend away trials (drug^∗^attention interaction: p < 0.001, two-factor ANOVA, Figure 7), and it even slightly speeded up the animal on attend RF trials (post hoc testing showed that RTs on attend RF CNQX applied trials were faster than on not applied trials, p = 0.042, t test).

## Discussion

Our results show that NMDA receptor availability is critical for attention-induced variance and noise correlation reduction, but not for attention-induced rate modulation in V1. Additionally, NMDA receptors play a key role in enabling attention-induced reduction of LFP gamma power, and they aid speedy reaction times. These effects were not simply a consequence of reduced excitatory drive, as blockade of AMPA/kainate receptors, yielded very different results, despite yielding a slightly stronger reduction in excitatory drive. Whereas AMPA/kainate receptors showed a trend toward playing a role in attention-induced rate variance reduction, they are not involved in attention-induced noise correlation reduction. Moreover, NMDA and AMPA/kainate receptor blockade had opposite effects on oscillatory activity in the gamma frequency range and on an animal’s reaction times. The effects reported were consistent for the two monkeys (Figures S2–S5).

A recent study (Self et al., 2012) reported that NMDA receptor availability is crucial for figure ground-induced rate modulations in macaque V1, whereas AMPA receptor availability is not. Figure ground rate modulation in V1 depends on feedback from higher areas (Hupé et al., 1998). Because it is assumed that top-down spatial attention in area V1 equally depends on feedback from higher areas, we expected that attention-induced rate modulations also depend on NMDA receptor availability. However, that was not the case. There are at least two possibilities that could account for the difference found. Self et al. (2012) used laminar multielectrodes to record neuronal activity from V1 layers 1–6 and performed single pressure injections at the border between layer 4 and layer 5. Given the approach, the largest drug efficacy would be in layers 5 and 4, and activity changes in layers 4 and/or 5 would invariably impact on the activity in other layers. Consequentially, drug effects seen in supragranular layers could be inherited from activity changes in lower layers. In our study, iontophoretic drug application was more directly targeted at the neurons recorded, as the pipette openings were within 20–40 μm of the electrode tip. Another factor possibly contributing to the different results were differences in the experimental approach. Self et al. (2012) did not systematically manipulate attention, but manipulated the visual stimulus instead, to yield differential neuronal activity. Conversely, in our study, activity difference arose from systematically manipulating continuous spatial top-down attention. Because both types of activity differences are assumed to be mediated by some form of feedback, it would imply that figure-ground-induced feedback signals exploit a different type of connections than attentional signals do, but that remains to be tested.

We found that attention-induced variance and noise correlation reduction appear to be general phenomena that occur not only in extrastriate (Cohen and Maunsell, 2009; Mitchell et al., 2007, 2009; Niebergall et al., 2011) but also in striate visual cortex. The finding that rate variance is strongly dependent on excitatory drive is expected, as the sudden appearance of a stimulus reduces rate variance (Churchland et al., 2010). Thus, blockade of AMPA/kainate receptors, should increase FFs for both attention conditions, which it did. However, a more selective effect was induced by NMDA receptor blockade. Whereas the attention-induced FF reduction was reduced upon NMDA receptor blockade, FFs did not increase beyond the level seen in the attend away control condition. This dissociation shows that the two receptors make different contributions to controlling rate variance. AMPA/kainate receptor availability is important for increased response reliability in the absence and presence of attention, whereas NMDA receptors selectively aid increased response reliability when attention is directed to the neuron’s RFs. Given that attention is usually linked to feedback signals from higher cortical areas (Buschman and Miller, 2007; Gregoriou et al., 2009; Moore and Armstrong, 2003; Noudoost and Moore, 2011; Roelfsema et al., 1998; Ruff et al., 2006) and/or the pulvinar (Saalmann et al., 2012), it suggests that attention-dependent feedback connections are especially reliant on NMDA receptors that control response fidelity but not response gain.

The differences between NMDA versus AMPA/kainate receptor blockade on neuronal activity were even more pronounced when analyzing noise correlations. Noise correlations in our study were larger than those reported recently by Ecker et al. (2010), but they were within the range reported by others (Bair et al., 2001; Gutnisky and Dragoi, 2008; Hansen et al., 2012; Kohn and Smith, 2005; Nauhaus et al., 2009), and they were significantly reduced by attention. Only NMDA receptor blockade abolished attention-induced noise correlations. Attention-induced reduction in rate covariance is an efficient way to increase the signal-to-noise ratio when decoding the activity from pools of neurons (Abbott and Dayan, 1999; Cohen and Kohn, 2011; Mitchell et al., 2009; Shadlen and Newsome, 1998), although this depends on the decoding strategy and decoding aims (Averbeck et al., 2006; Oram et al., 1998; Poort and Roelfsema, 2009). Specifically, reduction in rate covariance aids decoding when pooling across neurons with large signal correlations, but is less beneficial, or even detrimental, when activity from neurons is analyzed that have low signal correlations (Averbeck et al., 2006; Oram et al., 1998; Poort and Roelfsema, 2009). Neurons in our sample were recorded from the same electrode, and given the columnar organization of V1, they presumably had large signal correlation. Thus, attention-induced reduction of noise correlation would yield decoding benefits. Mitchell et al. (2009) showed that the attention-induced change of noise correlation in V4 had a substantially larger impact on the population signal-to-noise ratio than attention-induced changes of firing rates had. In this context, our data demonstrate that NMDA receptors contribute in very important ways to attentional modulation, even if they do not affect the rate changes. AMPA/kainate receptor blockade increased noise correlations, regardless of where the animal attended to, and the overall increase was similar to the increase of FFs reported above. Excitatory drive mediated by AMPA/kainate receptors thus stabilizes not only rate variance but also rate covariance, but it does not contribute to attention-induced reductions of noise correlations.

Attention reduced the power of gamma frequency oscillations in V1, as reported previously (Chalk et al., 2010). Blockade of NMDA receptors increased LFP gamma power, and it abolished the attention-induced reduction. This was accompanied by an increase in RT when the animals attended to the RF (and locus of drug application), as if the animals’ attention was reduced. Although it is tempting to speculate that these two phenomena are directly linked, it is likely an oversimplification, which does not do justice to the multitude of effects we have reported. After all, NMDA receptor blockade also affected FFs and noise correlations, which will also contribute to the changes in RTs. AMPA/kainate receptor blockade reduced the LFP gamma power, which somewhat mimicked the conditions of “attend RF” on attend away trials, and AMPA/kainate receptor blockade was accompanied by increased RTs on attend away trials during drug application and slightly decreased RTs in attend RF trials. It is equally tempting to link the CNQX-induced reduction in gamma power to changes in RT (faster on attend RF trials, slower on attend away trials), but as for the NMDA receptor blockade, CNQX affected a multitude of coding parameters, and it is unlikely that the effects at the behavioral level can be explained by just one of them.

Blockade of neither of the two receptors affected attention-induced gain changes, which depend on cholinergic mechanisms in V1 (Herrero et al., 2008). Conversely, ACh did not affect firing rate variance as measured by the FF (Herrero et al., 2008). We thus show a double dissociation between the effects of different transmitter systems (ACh versus glutamatergic) on mean rate and rate variance. But, even within the glutamatergic system a dissociation occurred at the level of attention-induced reduction of noise correlation, the level of LFP gamma oscillations and the level of reaction times. Attention-induced noise correlation reduction was only affected by NMDA blockade, not by AMPA/kainate blockade. We currently do not know whether the cholinergic system plays a role in attention-induced noise correlation reduction. It may do so, as muscarinic receptors in V1 are important to induce noise correlation reduction upon basal forebrain stimulation under anesthesia (Goard and Dan, 2009), but whether this also occurs in attending animals, remains to be established.

### Conclusions

NMDA receptors are critically involved in a variety of cognitive functions and might be critically involved in schizophrenia (Coyle et al., 2003; Roopun et al., 2008; Rowland et al., 2005). Modeling suggests that relatively high NMDA/AMPA receptor ratios are required to achieve realistic FF in large-scale network models (Rasch et al., 2011), and their role in the generation of persistent neuronal activity during working memory has been discussed repeatedly (Brunel and Wang, 2001; Compte et al., 2000; Wang, 2001). Working memory and top-down attention are likely to be related concepts, as a spatial top-down attention signal requires the location of interest to be constantly monitored and thus kept in memory. Our data show that continuous attentional feedback signals equally depends on a high NMDA/AMPA receptors ratio to achieve signal stability. In conjunction with our previous results regarding the role of acetylcholine in attentional modulation in V1, these data show that cognitive functions, such as attention, can be dissociated at the receptor level.

## Experimental Procedures

Two male rhesus monkeys (6–9 years old) were used for the electrophysiological recordings reported in this study. After initial training, monkeys were implanted with a head holder and recording chambers above V1 under general anesthesia and sterile conditions (for details of surgical procedures, postsurgical analgesics, and general postsurgical treatment, see Thiele et al., 2006). All procedures complied with the European Communities Council Directive RL 2010/63/EC, the U.S. National Institutes of Health Guidelines for the Care and Use of Animals for Experimental Procedures, and the UK Animals Scientific Procedures Act.

### Electrophysiological Recordings and Drug Application

A tungsten-in-glass electrode flanked by two pipettes was used for the recordings (Thiele et al., 2006). Drugs were applied iontophoretically through these pipettes (NeuroPhore BH-2, Digitimer, Welwyn Garden City, Hertfordshire, England). Pipette opening diameter varied between 1–4 μm. Pipette resistance varied between 12–150 MΩ, with most recordings at 20–75 MΩ. Hold currents for NMDA and APV were usually +10 nA, whereas it was +6 nA for CNQX. In rare occasions, when the pipette resistance was 10–20 MΩ, hold current was larger (e.g., +40 nA for NMDA). Ejection currents were usually −5, −6, and −7 nA for NMDA, APV, and CNQX, respectively. We did not obtain a drug-response curve for the neurons recorded, as this would require too much time before the main experimental paradigms with the risk of losing the cells before finishing the experiments. We aimed to yield relatively small but measureable drug effects. We therefore kept the ejection current at low levels. For the APV and CNQX experiments, this was done to prevent silencing the cells altogether (or too much), as this would preclude the analysis of attentional modulation of first- and second-order spiking statistics. For the case of NMDA application, we additionally wanted to keep the NMDA concentration at moderate levels to avoid glutamate toxicity or saturated neuronal responses. All drugs (Sigma-Aldrich) were dissolved in distilled water, and their concentrations and pH were 20 mM NMDA (pH 8.0), 20 mM APV (pH 8.0), and 1 mM CNQX (pH 8.5). Pipette-electrode combinations were inserted into V1 through the dura on a daily basis without the use of guide tubes. The integrity of the electrode and the pipettes were checked under the microscope before and after the recording sessions, in addition to measurements of the pipette impedance made before and after the recording at each recording site. Drug application was continuous during blocks of “drug applied.” The duration of each block could vary depending on the speed and accuracy with which the animal worked. On average drug application for each block was ∼10 min. For the data analysis, we removed the first 10–20 trials after a switch from no-drug to drug applied, as well as after a switch from drug applied to no-drug conditions. This was done because drug effects and recovery usually occurred with a slight delay of ∼0.5–2 min. We regularly compensated for the change in current during the ejection condition by increasing the hold current of one of the two pipettes, thereby keeping the overall current identical between the “hold” and “eject” conditions. This ensured that overall current level between “hold” and “eject” were identical, and therefore none of the effects described in the paper can be due to direct current effects. In addition to this control, hold and ejection currents and pH for the three drugs were basically identical, whereas the effects on firing rate, rate variance, noise correlation, LFP gamma power, and the animals’ behavior differed radically. This is testament that the effects were specific for the drug used and were not due to unspecific confounds.

Neurons were further analyzed if at least ten trials per condition were available. For the large majority of recordings, we obtained between 20–40 trials per attention and drug condition (after removal of “transition trials,” see above). The median number of trials for our APV recordings were n = 18 per condition (25^th^, 75^th^ percentiles: n = 16, n = 23). For NMDA recordings, the median number of trials was n = 22 per condition (25^th^, 75^th^ percentiles: n = 16, n = 34). For CNQX recordings, the median number of trials was n = 39 per condition (25^th^, 75^th^ percentiles: n = 36, n = 40).

### Data Collection

Stimulus presentation and behavioral control was managed by Remote Cortex 5.95 (Laboratory of Neuropsychology, National Institute for Mental Health, Bethesda, MD, USA, http://dally.nimh.nih.gov). Neuronal data were collected by Cheetah data acquisition (Neuralynx) interlinked with Remote Cortex. The waveforms of all spikes that exceeded a threshold set by the experimenter were sampled at 30 kHz. Spike data from the recording electrode were obtained by band-pass filtering the raw signal from 600–9,000 Hz. To obtain single unit data, offline sorting of these spike samples was carried out based on waveform features (Neuralynx spike sorting software and AlSort, a custom-based script). The LFP signal was band-pass filtered between 1–200 Hz (using a third-order Butterworth filter) and sampled continuously at 1 kHz.

### Behavioral Task and Stimuli

#### RF Mapping and Orientation Tuning Determination

At the beginning of each recording, receptive fields were mapped using a reverse correlation technique described previously (Gieselmann and Thiele, 2008). The RFs recorded in the current paper had an eccentricity of 2.9°–5.4°, with the majority at ∼4.0°. Orientation tuning was also determined by a reverse correlation technique as described previously (Gieselmann and Thiele, 2008). RF location and preferred orientation were determined online. Bar stimuli used in the main task were presented centered on the RF, and the bars were presented at the preferred orientation (see below).

#### Main Task

The task is outlined in Figure 1. A trial was initiated by holding a touch bar and fixating a red fixation point (FP, 0.1° diameter) presented centrally on a 20” analog CRT monitor (110 Hz, 1,600 × 1,200 pixels, 57 cm from the animal) on a gray background (21 cd/m^2^). A cue (blue annulus, 0.24° outer diameter, 0.18° inner diameter) was presented for 400 ms on one side of the fixation spot. The location of the cue indicated the location to which the monkey had to covertly attend. The cue was presented displaced along the axis connecting the FP and the RF location by one-quarter of the eccentricity of the neuron’s RF. The cue was displaced either toward or away from the RF to indicate whether attention should be directed toward or away from the stimulus presented in the RF. After cue offset, a 900 ms blank period occurred with just the FP present. Thereafter, two identical stimuli were presented (test stimuli), one centered on the RF and the other at the same eccentricity in the opposite hemifield. Spatial and temporal separation of the cue from the test stimuli ensured that it had no direct effect on the neuronal response to the test stimulus. Test stimuli were bars of preferred orientation and size (0.8° × 0.2°) at a luminance contrast of 40%–50% (Michelson contrast), which were darker than the homogenous gray background (i.e., at a luminance of 7–9 cd/m^2^). After 500–800 ms (randomized in 1 ms steps), a brighter patch (0.1° square) appeared at the center of one of the bars. If presented in the cued location, it is referred to as “target”; if presented in the uncued location, it is referred to as “distracter.” The target or distracter was brighter than the test stimuli by 5–7 cd/m^2^. After the presentation of a target, the monkey had to release the touch bar within 500 ms to receive a juice reward. If a distracter was presented first, the monkey had to continue to hold the touch bar and maintain fixation until target appearance. This occurred 1,000–1,300 ms (randomized in 1 ms steps) after the distracter appeared. If the monkey made no response, the trial was terminated 500 ms after presentation of the target or distracter, whichever appeared last. Premature (or incorrect) releases of the touch bar or failure to maintain fixation resulted in immediate trial termination. Correct touch bar releases also resulted in trial termination such that the monkey could have his reward and get ready to perform the next trial. Eye movements were recorded by an infrared based system (Thomas Recording, temporal resolution 220 Hz, spatial resolution 2.5′). Eye position during all trials was restricted to be within ±0.5°–0.7° of the fixation point.

We recorded activity from 451 neurons in two monkeys (203 in monkey 1 and 248 in monkey 2) in the presence and absence of different glutamatergic antagonist/agonist. APV was tested in 207 neurons; NMDA was tested in 87 neurons; and CNQX was tested in 157 neurons. For each neuron recorded, we ensured that recovery following drug application was adequate, and therefore we performed a t test, to determine whether neuronal activity significantly differed between initial recording and recovery periods. If it did differ, the neuron was excluded from further analysis. We also determined whether slow activity drifts occurred over time by calculating the correlation coefficient associated with single trial activity (within the window of interest) against trial number (i.e., time). This was done separately for the four different conditions (attend away/attend RF and drug/no drug). If the p value associated with the correlation was smaller than 0.05/4 = 0.0125, we concluded that activity was not stable over time, and the cell was excluded from further analysis. Finally, we also inspected whether rapid stepwise changes occurred during any point in time by careful visual inspection of the spike raster plots for every cell. If a sharp temporal instability occurred, we concluded that the requirement for stationarity was violated, and the cell was excluded from further analysis. Finally, we ensured that neurons were active during the sustained response period, as otherwise attention rate modulation indices (MI), ROCs, Fano factors (FF), and noise correlation calculation would not be very meaningful. The response was considered adequate if the minimum rate encountered in all of the four conditions (attend away-no drug; attend RF-no drug; attend away-drug; attend RF-drug) exceeded 5 Hz in the 200–500 ms window after stimulus onset and the rate in that window was significantly greater than the firing rate in the 300 ms preceding stimulus onset (Wilcoxon signed-rank test, p < 0.05). A minimum response of 5 Hz was used as a criterion in previous investigations of similar kind (Mitchell et al., 2007, 2009), and we adopted this criterion to aid quantitative comparison.

### Analysis of Attention and Drug Effects on Rate Variability

We calculated the number of spikes per trial (spike count) in the response window from 200–500 ms after stimulus onset for the four conditions (attend away-no drug; attend RF-no drug; attend away-drug; attend RF-drug) and determined whether attention or drug had an effect on the rate variability by calculating the Fano factor (FF). The FF was calculated according to:

$$FF=\frac{variance\left(spike\;count\right)}{mean\left(spike\;count\right)}.$$

### Noise Correlation Analysis

To analyze noise correlations, we required that both neurons recorded simultaneously from the same electrode fired at >5Hz during the analysis window of 200–500 ms after stimulus onset and that the response in both neurons during this analysis period was significantly greater than their firing rate in the 300 ms preceding the stimulus onset (Wilcoxon signed-rank test, p < 0.05). Prior to computing the noise correlations, we equated for firing rate differences between different attention and drug conditions and also controlled for potential rate fluctuations that occurred in both channels as time progressed (see sections above and the following). Following initial cells exclusion (see above), the implemented controls were identical to those described in a previous publication by Mitchell et al. (2009). It was, however, necessary to adopt their MATLAB code slightly (http://www.snl.salk.edu/∼jude/neuron_exchange/index.html), to take into account (and control for) attention-induced firing rate differences, as well as control for drug-induced firing rate differences. Specifically, we performed the following. (1) Given that our pharmacological manipulations induced changes in firing rates, we equated firing rates in drug and no-drug conditions before calculating correlation estimates. The adjustment of firing rates for the different drugs was done in a manner identical to the adjustment of attention-induced firing rates described in Mitchell et al. (2009). The same procedure was done for the different attention conditions. (2) We also eliminated trends of rate changes that may be shared across neurons. This trend removal included (1) removal of fluctuations in rate that occur within trials (i.e., consistent changes in firing between units that are time locked to events). This was done by subtracting out the mean response for each condition and for each neuron from the single trial response and (2) fluctuations in rate that span trials (e.g., drifts over long timescales in an experiment). The latter was done by subtracting out the mean firing rate smoothed over adjacent trials using a Gaussian smoothing window with a width of five trials. This smoothed firing rate was then subtracted from the spike counts of each trial to give normalized spike counts in a manner identical to established procedures (Bair et al., 2001; Cohen and Newsome, 2009; Mitchell et al., 2009). We then calculated a Spearman rank correlation coefficient between the trial wise normalized spike counts from the two neurons for each of the four conditions (attend RF no drug; attend RF drug; attend away no drug; attend away drug). We Fisher transformed the correlation coefficients and then performed a two-factor repeated-measurement ANOVA (factor 1: drug applied/not applied; factor 2: attention) on the data to determine whether attention or drug had a significant effect on noise correlations and whether there were any interactions. Although the above analysis should take care of slow fluctuations, we performed additional controls, by calculating the shift predictor of the noise correlations and subtracted these values from the raw noise correlations. This was done in two different ways. First, the shift predictor was calculated by using trial 1 to trial n − 1 in cell 1 and calculated the noise correlation with cell two using trial 2 to trial n (i.e., 1 trial offset in a continuous manner), and finally we used trial n from cell 1 with trial 1 from cell 2. This procedure could still be affected by slow drifts. We therefore also calculated the shift predictor by using trial 1 to trial n from cell 1 and randomly selected (without replacement) a trial from cell 2 to calculate the shift predictor (whereby the trial number selected for cell 1 was always different from the trial number selected for cell 2). The resultant shift predictor noise correlations were subtracted from the raw noise correlations before Fisher transformation and statistical testing. The outcome of the shift predictor noise correlation analysis is reported in Figure S1.

### Analysis of LFP

For recordings to be included into the LFP and behavioral analysis, we required that the multiunit activity (MUA, activity before offline spike sorting was performed) recorded from the electrode showed a significant drug effect (or drug-attention interaction, p < 0.05) and that the MUA activity after drug application recovered to levels that were recorded prior to drug application, i.e., that good recovery occurred at the level of spiking activity. Here, we used the MUA activity as an inclusion criterion, as LFP and behavioral measures are likely based on larger neuronal ensembles, which we wanted to be influenced by the drug in the first place. Note that this does not pre-empt any effects on the LFP or behavior as the sign of effects on the MUA was irrelevant for the preselection.

LFP analyses were performed using multitaper technique (Percival and Walden, 1993), under the Chronux toolbox (http://www.chronux.org). We used a time-bandwidth product of TW = 2 with K = 3 tapers, with no padding. Because we were interested in the sustained response, we estimated the raw power spectral density of the single trial LFP response (RPS) over the time period of 256–511 ms after stimulus onset. For each recording site, the mean power spectrum (PSM) was calculated from the single-trial RPS data. We repeated the same procedure for the time period 255–0 ms before stimulus onset to obtain the mean baseline power spectrum (BPSM) and the SD of the baseline power spectrum (BPSSD). The stimulus-induced (Pz) power spectrum was then calculated as follows:

$$\text{Pz}=\frac{\left(\text{PSM}\text{-}\text{BPSM}\right)}{\text{BPSSD}}.$$

Pz was obtained for each attentional and drug condition in order to provide a measure of stimulus-induced spectral power. Induced power spectra (Pz) were subjected to a two-factor ANOVA (factor 1: drug applied/not applied; factor 2: attention). Additional details regarding the LFP analyses can be found in Chalk et al. (2010). We did not analyze high gamma frequency bands due to the documented risk of spike intrusion (Ray and Maunsell, 2011). Note that the analyses of different frequency bands in the low-frequency domain will be affected by spectral smear, and for the case of the delta frequency band, it will also be affected by the limited time period available (256 ms when analyzing the spectral power at 3.81 Hz [the bin used for the delta range]). Given the widely used subdivision into the delta, theta, alpha, beta, and gamma frequency bands, we nevertheless decided to present our data in that format, despite the caveats mentioned.

### Analysis of Behavioral Responses

Reaction times (RT) were monitored across different attention and drug conditions. Animals had to release a manual lever as soon as they detected a small contrast luminance change in the target bar, while ignoring luminance changes in the distracter bar. Lever releases faster than 50 ms were considered as incorrect responses, as well as releases slower than 500 ms. RTs were normalized by subtracting the mean RT associated with correct responses (obtained by averaging across all conditions from a daily recording session) from each individual trial RT. Normalized single trial RTs obtained from all the different sessions were then subjected to a single repeated-measures ANOVA, i.e., an ANOVA based on many thousands of trials. We normalized the RTs within a session to account for slight differences in RTs over different days that may be induced by different RF location (more eccentric locations would increase the level of difficulty) and possible differences in motivational levels, which are also likely to show slight fluctuations between days.

## Figures and Tables

**Table 1:** Cell Responses in the Three Data Sets—APV, NMDA, and CNQX—and for the Four Experimental Conditions

	APV (n = 82)	NMDA (n = 43)	CNQX (n = 96)
Attend away, no drug	36.4 ± 3.2	47.7 ± 6.1	63.6 ± 4.1
Attend RF, no drug	41.9 ± 3.6	52.1 ± 6.9	68.6 ± 3.9
Attend away, drug	24.8 ± 2.6	55.6 ± 6.9	43.7 ± 3.6
Attend RF, drug	28.1 ± 2.8	61.0 ± 7.6	46.5 ± 3.5

Mean (spikes/s) ± SEM. Time window used for the analysis was 200–500 ms after stimulus onset. n, number of cells in each data set.
