# Supplementary material for: Attention-Induced Variance and Noise Correlation Reduction in Macaque V1 Is Mediated by NMDA Receptors
Source: Neuron. 2013 May 22;78(4):729–39. doi: 10.1016/j.neuron.2013.03.029 (PMC3748348; doi:10.1016/j.neuron.2013.03.029)
Supplement: Document S1. Figures S1–S5 [file mmc1.pdf]

Neuron, Volume *78*

Supplemental Information

Attention-Induced Variance and Noise Correlation Reduction in Macaque V1 Is Mediated by NMDA Receptors

Jose L. Herrero, Marc A. Gieselmann, Mehdi Sanayei, and Alexander Thiele

Contents

Calculation of shift predictor corrected noise correlations 3

Figure S1 (related to Figure 5): 5

Drug effects analysed separately for the two monkeys 5

Drug effects on modulation indices, ROCs, and Fano-factors 5

Figure S2 (related to Figure 4): 6

Drug effects on noise correlations 7

Figure S3 (related to Figure 5) 8

Drug effects on LFP power 9

Figure S4 (related to Figure 6): 9

Drug effects on reaction times 10

Figure S5 (related to Figure 7): 10

Supplemental References 11

Calculation of shift predictor corrected noise correlations

The noise correlations reported in the main paper were calculated in a manner identical to that reported by Mitchell et al. (Mitchell et al., 2009), with additional tight controls for stationarity as reported in the methods section (main paper). However, it could still be the case that some of the noise correlation differences are due to non-stationarities in the data that may have escaped us. An additional way to control for this is by calculating shift predictor corrected noise correlations (Brody, 1999a, b). The shift predictor can be calculated by using the activity of neuron 1 in trial 1 to n-1 and correlating it with the activity of neuron 2 in trial n+1 to n (shift predictor correction method 1). This, could in principle still be prone to errors as very slow drifts in activity could still contribute to noise correlation values. Shift predictors calculated by taking trial 1 to n from neuron 1, and randomly selection a trial for neuron 2 (other than the trial order number that was used for neuron 1) should overcome that problem (shift predictor correction method 2). Here we calculated shift predictor corrected noise correlations (i.e. raw noise correlation minus the shift predictor noise correlation) for both types of correction (methods 1 and 2) and have obtained qualitatively identical results to those described in the main manuscript (compare Figure S1 to Figure 3 in the main manuscript).

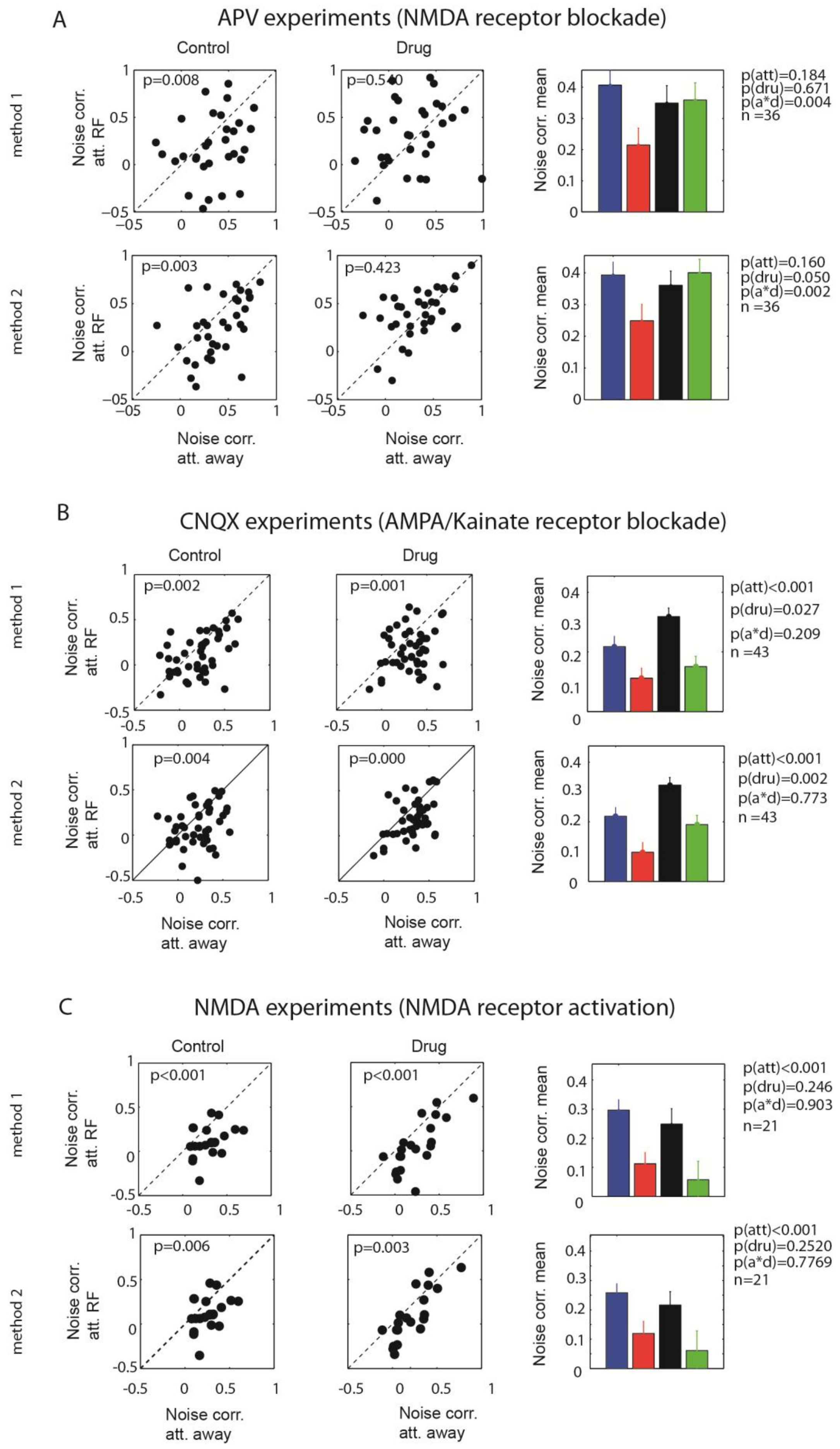
A
APV experiments (NMDA receptor blockade)
Control
Drug
method 1
method 2
Noise corr. att. RF
Noise corr. att. away
Noise corr. mean
p=0.008
p=0.540
p=0.003
p=0.423
p(att)=0.184
p(dru)=0.671
p(a*d)=0.004
n =36
p(att)=0.160
p(dru)=0.050
p(a*d)=0.002
n =36
B
CNQX experiments (AMPA/Kainate receptor blockade)
Control
Drug
method 1
method 2
Noise corr. att. RF
Noise corr. att. away
Noise corr. mean
p=0.002
p=0.001
p=0.004
p=0.000
p(att)<0.001
p(dru)=0.027
p(a*d)=0.209
n =43
p(att)<0.001
p(dru)=0.002
p(a*d)=0.773
n =43
C
NMDA experiments (NMDA receptor activation)
Control
Drug
method 1
method 2
Noise corr. att. RF
Noise corr. att. away
Noise corr. mean
p<0.001
p<0.001
p=0.006
p=0.003
p(att)<0.001
p(dru)=0.246
p(a*d)=0.903
n=21
p(att)<0.001
p(dru)=0.2520
p(a*d)=0.7769
n=21

Figure S1 (Related to Figure 5). Shift predictor corrected noise correlations when (A) APV, (B) CNQX, (C) NMDA was not applied and when it was applied . For an explanation of method 1 (top row in A, B, C, respectively) and method 2 (bottom row in A, B, C, respectively) see supplemental text. Noise correlations are shown as scatter plots and means +/- SEM (coloured bars) for the attend away [att.away], the attend RF [att. RF], the no drug [control] and the drug [drug] condition). P-values in scatter plots indicate significance based on Wilcoxon signed rank tests. P-values to the right of bar graphs indicate significance of effects based on a 2 factor repeated measures ANOVA (p(att)=main effect of attention; p(dru): main effect of drug; p(att*drug): interaction between attention and drug). Blue bars indicate the attend away/no drug condition, red bars indicate the attend RF/no drug condition, black bars indicate the attend away/drug applied condition, green bars the attend RF/drug applied condition. N=number of cell pairs.

Drug effects analysed separately for the two monkeys

Drug effects on modulation indices, ROCs, and Fano-factors

Attention reduced FFs in V1 in the 'no drug' condition. In the experiments where NMDA receptors availability was manipulated (APV applied, not applied), the effect of attention on Fano-factors depended on whether or not the drug was applied in both monkeys (significant interaction between attention and drug term, Figure S2). In the experiments where AMPA/Kainate receptors availability was manipulated (CNQX applied, not applied), the effect of attention on Fano-factors was not dependent on whether or not the drug was applied (no significant interaction between attention and drug term for either monkey, Figure S2). In the experiments where NMDA receptors activity was enhanced (NMDA applied, not applied), the effect of attention on Fano-factors was independent on whether or not the drug was applied (no significant interaction between attention and drug term for either monkey, Figure S2). Moreover, APV application did not systematically affect FFs overall (no drug main effect in either monkey), while CNQX application tended to increase FFs in both monkeys (this was only significant in monkey 1, with a trend towards significance in monkey 2 [p=0.072], Figure S2). NMDA application did not have a main effect on FFs in either monkey (Figure S2).

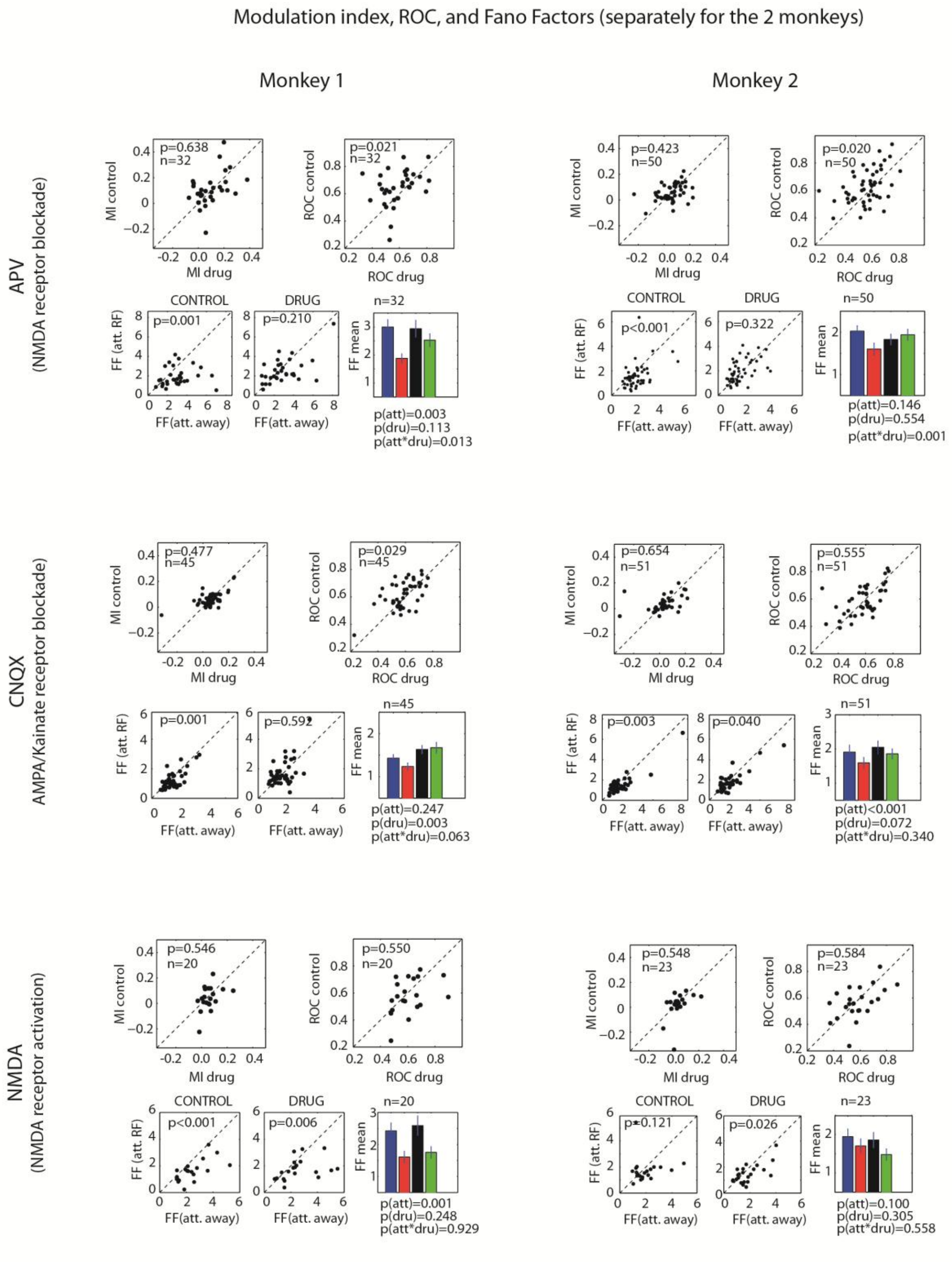

Figure S2 (Related to Figure 4). Effect of NMDA receptor blockade (top rows), AMPA/kainate receptor blockade (middle rows) and NMDA receptor activation (bottom rows) on modulation indices (MI, left upper scatter plot in subpanel groupings), receiver operating characteristic (ROC, right upper scatter plot in subpanel groupings) values, and Fano-factors (FF, scatter plots and means for the attend away [att.away], the attend RF [att. RF], the no drug [control] and the drug [drug] condition) for monkeys 1 (left columns) and 2 (right columns). P-values in scatter plots indicate significance based on Wilcoxon signed rank tests. P-values below bar graphs indicate significance of effects based on a 2 factor repeated measures ANOVA (p(att)=main effect of attention; p(dru): main effect of drug; p(att*drug): interaction between attention

and drug). Blue bars indicate the attend away/no drug condition, red bars indicate the attend RF/no drug condition, black bars indicate the attend away/drug applied condition, green bars the attend RF/drug applied condition.

Drug effects on noise correlations

Figure S3 shows the breakdown of drug effects on noise correlation separately for the two monkeys. Attention decreased noise correlations in both monkeys (compare blue and red bars in Figures S3 and corresponding scatter plots). The effect was significant ($p<0.001$) for each monkey when data for the no drug conditions were analysed individually, and when pooled across the 3 sets of experiments (see bottom row of Figures S3). Importantly, in both monkeys the effect of attention on noise correlation depended on the availability of NMDA receptors (significant interaction between attention and drug in the APV experiment in both monkeys, Figure S3), but it did not depend on the availability of AMPA/Kainate receptors (no interaction between attention and drug in the CNQX experiment in either monkey, Figure S3). Also, in contrast to NMDA receptor blockade [which did not have a main drug effect on noise correlations in either monkey], AMPA/Kainate receptor blockade increased noise correlations overall in each monkey (Figure S3). Finally, NMDA application did not alter attention-mediated reduction of noise correlations in either monkey (Figure S3).

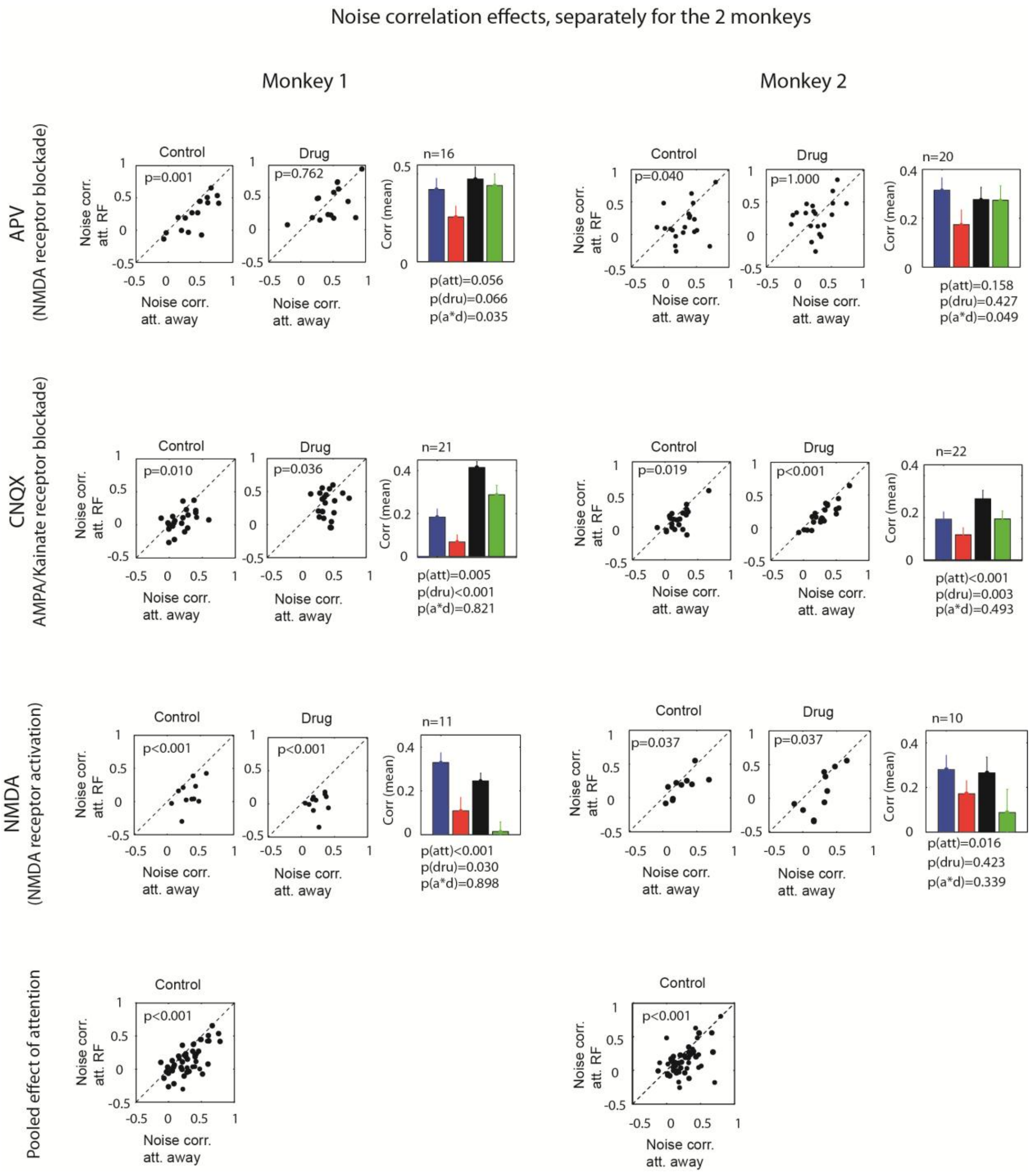

Figure S3 (Related to Figure 5). Effect of NMDA receptor blockade (top row), AMPA/kainate receptor blockade(second row) and NMDA receptor activation (third row) on noise correlations (scatter plots and means respectively for the attend away [att.away], the attend RF [att. RF], the no drug [control] and the drug [drug] condition) for monkeys 1 (left columns) and 2 (right columns)). P-values in scatter plots indicate significance based on Wilcoxon signed rank tests. P-values below bar graphs indicate significance of effects based on a 2 factor repeated measures ANOVA (p(att)=main effect of attention; p(dru): main effect of drug; p(att*drug): interaction between attention and drug). Blue bars indicate the attend away/no drug condition, red bars indicate the attend RF/no drug condition, black bars indicate the attend away/drug applied condition, green bars the attend RF/drug applied condition. The bottom row shows the effect of attention on noise correlation for the two monkeys when 'no drug' data are pooled across experiments.

Drug effects on LFP power

Figure S4 shows the breakdown of drug effects on LFP gamma z-score power separately for the two monkeys. Attention decreased LFP gamma power in both monkeys (compare blue and red bars in Figure S4), but this effect depended on whether or not APV was applied. In both monkeys the effect of attention on LFP gamma power depended on the availability of NMDA receptors (significant interaction between attention and drug in the APV experiment in monkey 2, and near significant interaction in monkey 1, Figure S4), but it did not depend on the availability of AMPA/Kainate receptors (no interaction between attention and drug in the CNQX experiment in either monkey, Figure S4). Moreover APV application increased LFP gamma power in both monkeys, while CNQX decreased LFP gamma power in both monkeys (Figure S4).

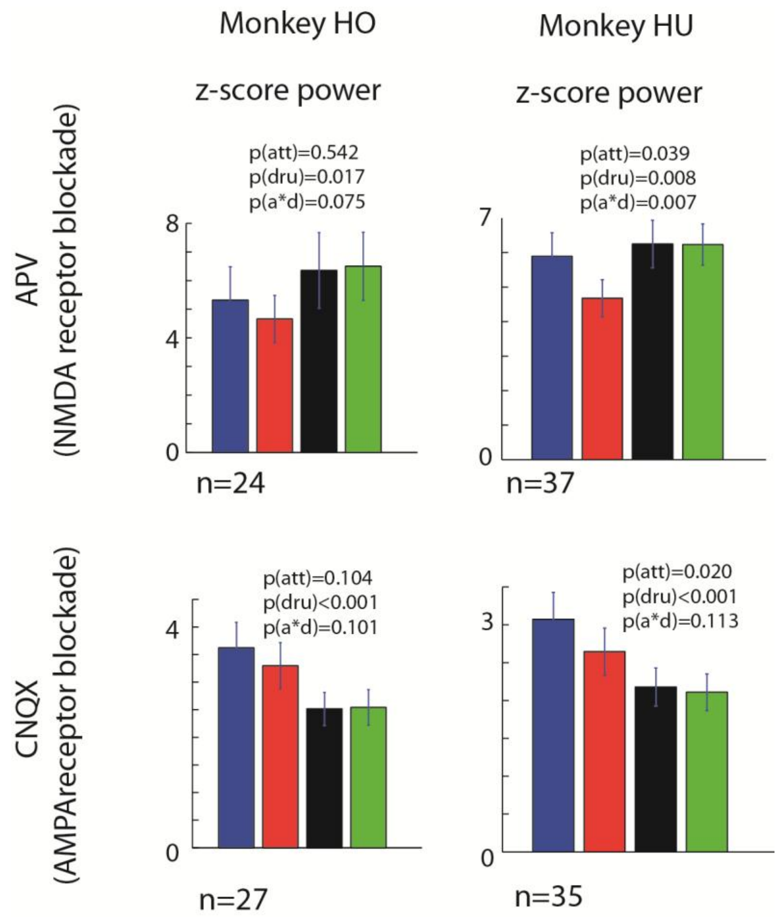

Figure S4 (Related to Figure 6). Effect of NMDA receptor blockade (top row), and AMPA/kainate receptor blockade (second row) on LFP gamma power (30-60 Hz, z-score) for monkeys 1 (left columns) and 2 (right columns). P-values above bar graphs indicate significance of effects based on a 2 factor repeated measures ANOVA (p(att)=main effect of attention; p(dru): main effect of drug; p(att*drug): interaction between attention and drug. Blue bars indicate the attend away/no drug condition, red bars indicate the attend RF/no drug condition, black bars indicate the attend away/drug applied condition, green bars the attend RF/drug applied condition. n=number of experiments performed in a given animal with the drug of interest.

Drug effects on reaction times

Figure S5 shows the breakdown of drug effects on normalized reaction times separately for the two monkeys. Application of APV increased reaction times in the attend RF condition (compare red and green graphs), which was significant in monkey 2 (significant interaction between attention and drug, 2 Factor ANOVA, Figure S5). CNQX increased reaction times in the attend away condition in both monkeys (compare blue and black bars, significant interaction between attention and drug, 2 Factor ANOVA, Figure S5).

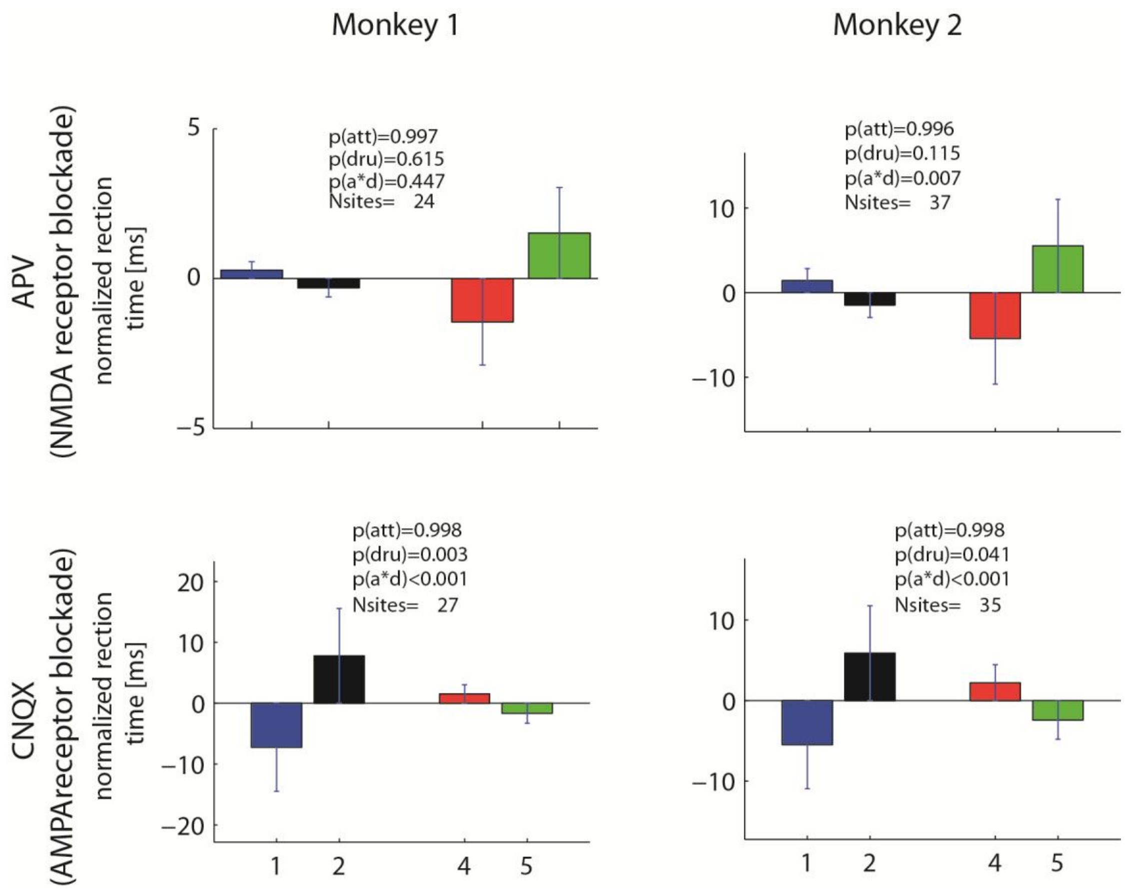

Figure S5 (Related to Figure 7). Effect of NMDA receptor blockade (top row), and AMPA/kainate receptor blockade (second row) on normalized reaction times for monkeys 1 (left columns) and monkey 2 (P-values above bar graphs indicate significance of effects based on a 2 factor repeated measures ANOVA (p(att)=main effect of attention; p(dru): main effect of drug; p(att*drug): interaction between attention and drug). Blue bars indicate the attend away/no drug condition, red bars indicate the attend RF/no drug condition, black bars indicate the attend away/drug applied condition, green bars the attend RF/drug applied condition. n=number of experiments performed in a given animal with the drug of interest. Nsites: number of experimental session with and without drug applied.

Supplemental References

Brody, C.D. (1999a). Correlations without synchrony. Neural Computation 11, 1537-1551.

Brody, C.D. (1999b). Disambiguating different covariation types. Neural Computation 11, 1527-1535.

Mitchell, J.F., Sundberg, K.A., and Reynolds, J.H. (2009). Spatial attention decorrelates intrinsic activity fluctuations in macaque area V4. Neuron 63, 879-888.
